# Identification of LSD analogs, 1cP-AL-LAD, 1cP-MIPLA, 1V-LSD and LSZ in sheet products

**DOI:** 10.1007/s11419-023-00661-1

**Published:** 2023-02-21

**Authors:** Rie Tanaka, Maiko Kawamura, Sakumi Mizutani, Ruri Kikura-Hanajiri

**Affiliations:** Division of Pharmacognosy, Phytochemistry and Narcotics, National Institute of Health Sciences, 3-25-26, Tonomachi, Kawasaki-Ku, Kawasaki, Kanagawa 210-9501 Japan

**Keywords:** Lysergic acid diethylamide, LSD, Lysergamide, Blotter paper, New psychoactive substance (NPS)

## Abstract

**Purpose:**

Many analogs of lysergic acid diethylamide (LSD) have recently appeared as designer drugs around the world. These compounds are mainly distributed as sheet products. In this study, we identified three more newly distributed LSD analogs from paper sheet products.

**Methods:**

The structures of the compounds were determined by gas chromatography–mass spectrometry (GC–MS), liquid chromatography–photodiode array–mass spectrometry (LC–PDA–MS), liquid chromatography with hybrid quadrupole time-of-flight mass spectrometry (LC–Q-TOF-MS) and nuclear magnetic resonance (NMR) spectroscopy.

**Results:**

From the NMR analysis, the compounds in the four products were identified as 4-(cyclopropanecarbonyl)-*N*,*N*-diethyl-7-(prop-2-en-1-yl)-4,6,6a,7,8,9-hexahydroindolo[4,3-*fg*]quinoline-9-carboxamide (1cP-AL-LAD), 4-(cyclopropanecarbonyl)-*N*-methyl-*N*-isopropyl-7-methyl-4,6,6a,7,8,9-hexahydroindolo-[4,3-*fg*]quinoline-9-carboxamide (1cP-MIPLA), *N,N*-diethyl-7-methyl-4-pentanoyl-4,6,6a,7,8,9-hexahydroindolo[4,3-*fg*]quinoline-9-carboxamide (1V-LSD) and (2’*S*,4’*S*)-lysergic acid 2,4-dimethylazetidide (LSZ). In comparison with the structure of LSD, 1cP-AL-LAD was converted at the positions at N1 and N6, and 1cP-MIPLA was converted at the positions at N1 and N18. The metabolic pathways and biological activities of 1cP-AL-LAD and 1cP-MIPLA have not been reported.

**Conclusions:**

This is the first report showing that LSD analogs that were converted at multiple positions have been detected in sheet products in Japan. There are concerns about the future distribution of sheet drug products containing new LSD analogs. Therefore, the continuous monitoring for newly detected compounds in sheet products is important.

**Supplementary Information:**

The online version contains supplementary material available at 10.1007/s11419-023-00661-1.

## Introduction

The regulation of a drug of abuse leads to the emergence of other compounds with partially altered structures. Consequently, new and unregulated synthetic cannabinoids, cathinones and fentanyl derivatives are continuously emerging. This so-called ‘cat-and-mouse’ game is still ongoing.

**Fig. 1:**
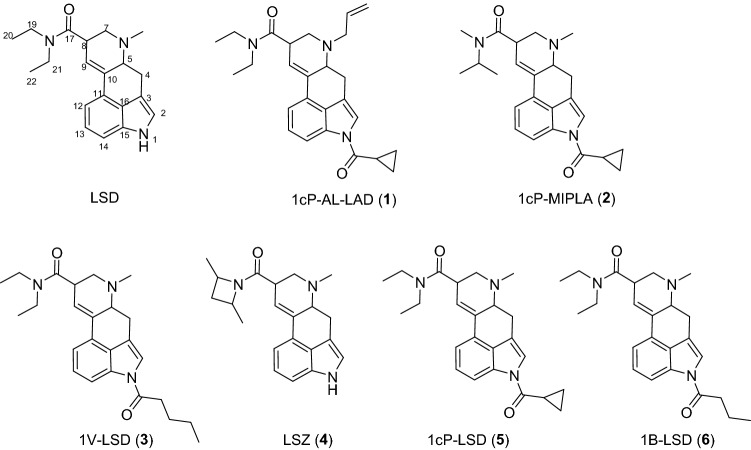
Chemical structures of LSD, 1cP-AL-LAD, 1cP-MIPLA, 1V-LSD, LSZ, 1cP-LSD and 1B-LSD

Recently, many lysergic acid diethylamide (LSD) [1] analogs have appeared as designer drugs throughout the world [2, 3]. These compounds are mainly distributed as sheet products. LSD analogs such as *N,N-7*,-triethyl-4,6,6a,7,8,9-hexahydroindolo[4,3-*fg*]quinoline-9-carboxamide (ETH-LAD) and 7-Allyl-*N*,*N*-diethyl-4,6,6a,7,8,9-hexahydroindolo[4,3-*fg*]quinoline-9-carboxamide (AL-LAD), in which the methyl group at the N6 position of LSD is changed, and 4-Acetyl-*N,N*-diethyl-7-methyl-4,6,6a,7,8,9-hexahydroindolo[4,3-*fg*]quinoline-9-carboxamide (ALD-52), *N,N*-diethyl-7-methyl-4-propionyl-4,6,6a,7,8,9-hexahydroindolo[4,3-*fg*]quinoline-9-carboxamide (1P-LSD), 4-cyclopropionyl-*N*,*N*-diethyl-7-methyl-4,6,6a,7,8,9-hexahydroindolo[4,3-*fg*]quinoline-9-carboxamide (1cP-LSD) and 4-butyryl-*N*,*N*-diethyl-7-methyl-4,6,6a,7,8,9-hexahydroindolo[4,3-*fg*]quinoline-9-carboxamide (1B-LSD), in which the N1 position is acylated, have been reported [4–10] (Fig. 1). It has been reported that deacylation of these N1-acylated LSD analogs occurs in vivo [11]. Recently, a new N1-acylated LSD analog, *N,N*-diethyl-7-methyl-4-pentanoyl-4,6,6a,7,8,9-hexahydroindolo[4,3-*fg*]quinoline-9-carboxamide (1V-LSD), was reported, and head-twitch response studies of 1V-LSD in mice have been reported to show a dose-dependent increase similar to that of LSD [12].

In this paper, we describe the analyses and identification of four LSD analogs in from paper sheet products obtained between 2021 and 2022 in Japan.

## Materials and methods

### Chemicals

HPLC-grade acetonitrile and methanol were purchased from Kanto Chemical Co., Inc. (Tokyo, Japan). Methanol-*d*_4_ (99.8 at. % D) was purchased from Isotec, Inc. (Miamisburg, OH, USA). LSD was purchased from Cerilliant Corporation (Round Rock, TX, USA). (2’*S*,4’*S*)-Lysergic acid 2,4-dimethylazetidide (LSZ) was purchased from Chiron (Trondheim, Norway). Bond Elut C18, 500 mg, 3 mL (Agilent, Santa Clara, CA, USA) was used for purification.

### Samples

The four products (A to D) analyzed in this study were obtained in Japan between August 2021 and March 2022. Each sheet was a square with an overall size of 4.5 × 4.5 cm and perforated in a grid pattern with approximately 9 mm on each side. The structural formula of the LSD analog suggested to be contained was printed on one side of the sheet, and the name of the compound was printed on the reverse side.

### Sample preparation

Eight mg of each sheet cut into 2 mm squares was extracted with 1 mL of methanol in 15 min under sonication. Methanol was removed from the extract under a nitrogen stream and redissolved in 1 mL of acetonitrile for analysis by gas chromatography–mass spectrometry (GC–MS), liquid chromatography–photodiode array–mass spectrometry (LC–PDA–MS) and liquid chromatography with hybrid quadrupole time-of-flight mass spectrometry (LC–Q-TOF-MS). For nuclear magnetic resonance (NMR) spectroscopy, each piece of a cut sheet was sonicated in 1.0 mL of methanol at room temperature for 5 min. The extraction procedure was repeated three times. The extracts were combined and the solvent was removed using an evaporator. Each residue was purified with Bond Elut C18 eluted with water–methanol, dissolved in 0.3 mL of methanol-*d*_4_, and then subjected to NMR spectroscopy.

### LC–PDA–MS conditions

The LC–PDA–MS analysis was performed on an ACQUITY UPLC system with a mass detector and a photodiode array (PDA) detector (Waters, Milford, MA, USA). Chromatographic separation was performed using an ACQUITY HSS T3 (2.1 mm i.d. × 100 mm, 1.8 μm particle size, Waters) UPLC column protected by Van Guard HSS T3 (2.1 mm i.d. × 5 mm, 1.7 μm particle size, Waters) at 40 °C. For the LC–MS analysis, we employed a binary mobile phase of solvent A (0.1% formic acid in water) and solvent B (0.1% formic acid in acetonitrile). The samples were analyzed by the following elution program: 5–20% B (0–20 min) and then up to 80% B (20–30 min, 10-min hold) at a flow rate of 0.3 mL/min. The injection volume was 1 μL and the wavelength of the PDA detector for screening was set from 210 to 450 nm. The MS conditions for the LC–ESI-MS were as follows: positive and negative ionization, nitrogen desolvation gas (flow rate 650 L/h at 350 °C), capillary and cone voltages of 2500 V and 30 V, respectively, and mass spectral range *m/z* 120–650 [9, 10].

### GC–MS conditions

The GC–MS was performed on an Agilent 6890 N GC system with a 5975 mass-selective detector (Agilent Technologies, Santa Clara, CA, USA) using a capillary column (DB-1HT capillary, 15 m × 0.25 mm i.d., 0.10-μm film thickness; Agilent Technologies) with helium-gas carrier flowing at 1.0 mL/min. The conditions were as follows: electron energy, 70 eV; injector temperature, 200˚C; injection mode, splitless mode for 1.0 min; transfer line temperature, 280˚C; scan range, *m/z* 40–550. The oven temperature was held at 120˚C for 1 min, and then increased at 15˚C/min to 280˚C, where it was held for 5 min [9, 10].

### HR-MS analysis conditions

The HR-MS analysis was carried out on a TripleTOF^®^ 6600 LC/MS/MS system (AB SCIEX, Framingham, MA, USA) and a Nexera X2 system (Shimadzu, Kyoto, Japan). Chromatographic separation was performed in an ACQUITY HSS T3 (2.1 mm i.d. × 100 mm, 1.8 μm particle size, Waters) UPLC column protected by Van Guard HSS T3 (2.1 mm i.d. × 5 mm, 1.7 μm particle size, Waters) at 40 °C. The mobile phase was a binary phase of solvent A (0.1% formic acid in water) and solvent B (0.1% formic acid in acetonitrile) with a gradient program of A/B 95/5–5/95 (10 min, 2 min hold). The flow rate was 0.3 mL/min, and the elution was monitored at 210–450 nm. The mass spectrometer was operated in ESI mode with an ion-spray voltage of 5500 V (positive mode), a source temperature of 550 °C, an ion-source gas of nitrogen, a pressure of 50 psi for ion-source gases 1 and 2, a curtain gas pressure of 25 psi, and a declustering potential of 80 V. The samples were analyzed in TOF-MS scan mode (*m/z* 100–650) [9, 10].

### NMR spectrometry and parameters

The ^1^H-NMR and ^13^C-NMR spectra were measured using a JEOL JMN-ECA800 or ECZ800 spectrometer (JEOL, Tokyo, Japan). The chemical shifts were presented with reference to the residual deuterated methanol (CD_3_OD, *δ*_H_ 3.33 ppm and δ_C_ 49.0 ppm) in the NMR. Compound identification was performed using ^1^H-NMR, ^13^C-NMR, heteronuclear multiple quantum coherence (HMQC), heteronuclear multiple bond correlation (HMBC), H–H correlation spectroscopy (H–H COSY) and nuclear Overhauser effect spectroscopy (NOESY).

## Results

### Analysis of sheet product A

**Fig. 2:**
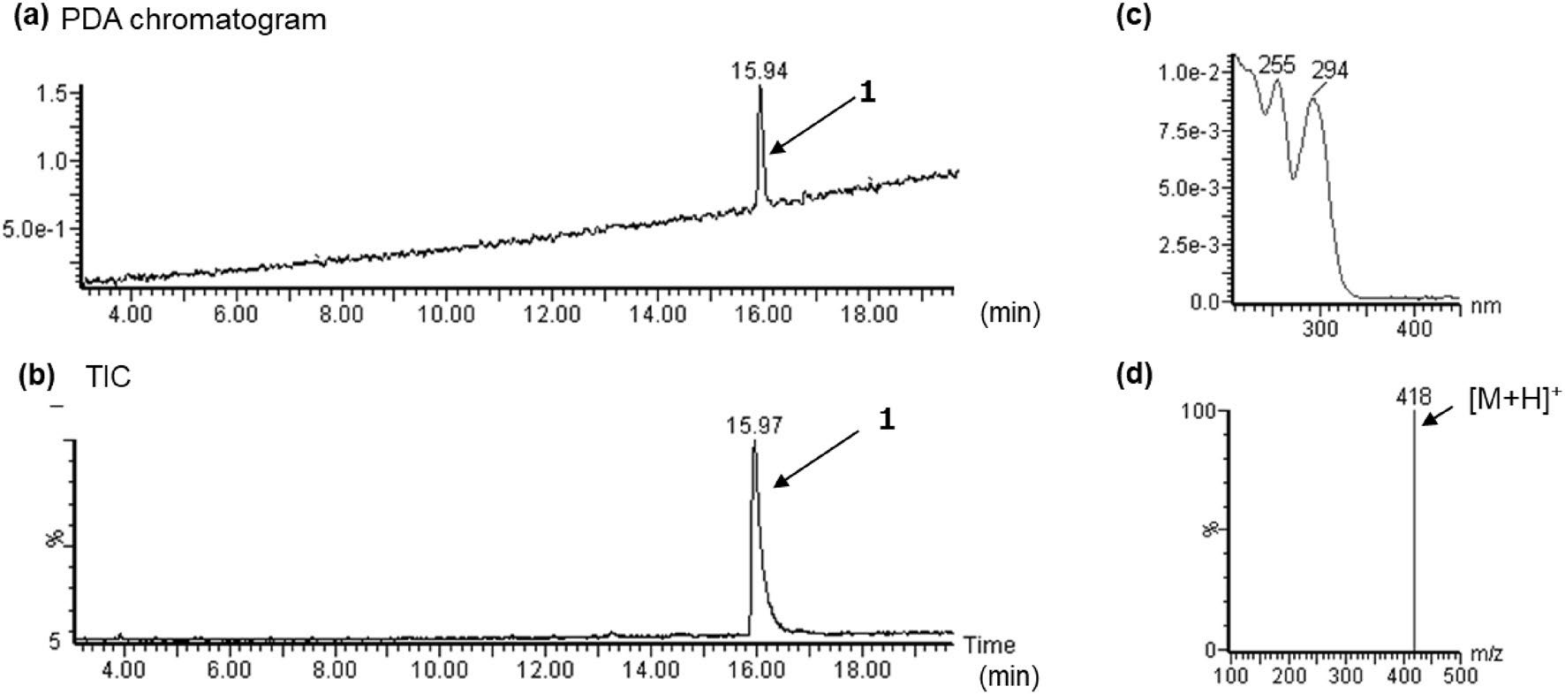
LC–PDA–MS analysis of sheet A; PDA chromatogram **a**, TIC **b**, and UV and ESI mass spectra of peak 1 **c**, **d**

**Fig. 3:**
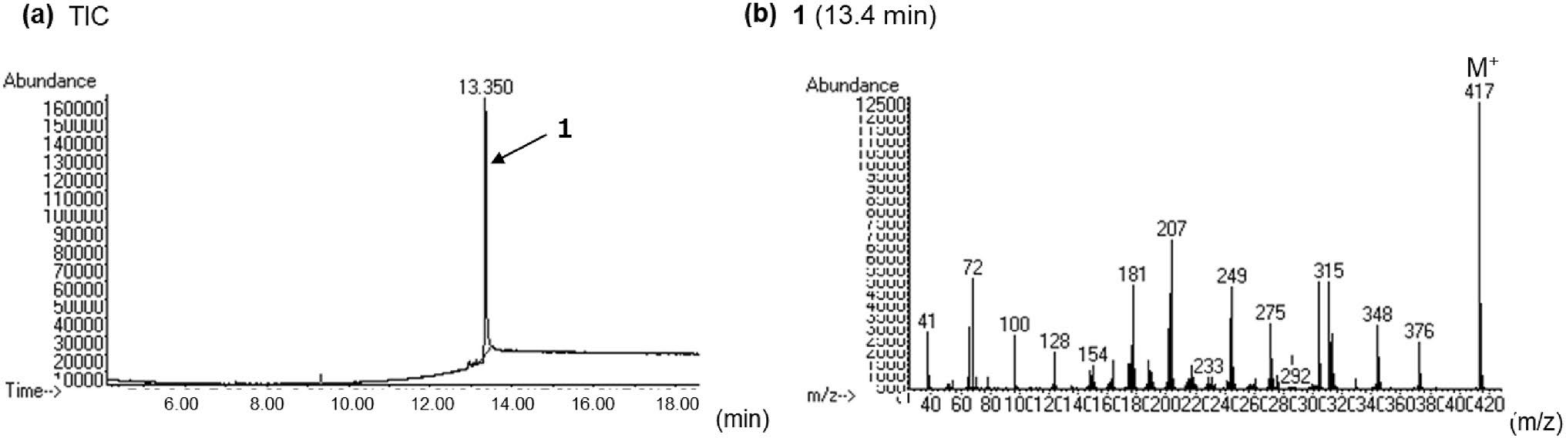
GC–MS analysis of sheet A; TIC **a**, EI mass spectrum of peak 1 **b**

**Fig. 4:**
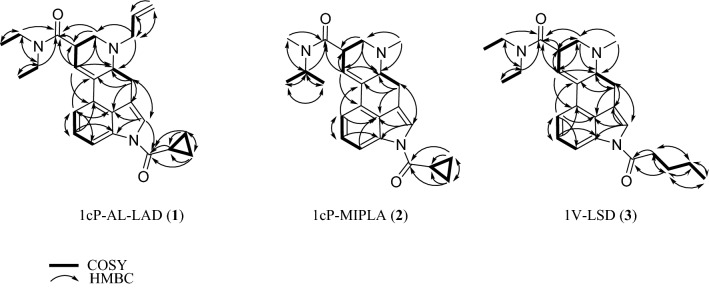
COSY and HMBC correlations of LSD analogs

In the LC–PDA–MS analysis of product A, the peak for compound **1** was detected at 16.0 min with a protonated molecular ion ([M + H]^+^) at *m/z* 418 (Fig. 2a and b). In the GC–MS analysis, the peak at 13.4 min indicated a molecular ion ([M]^+^) at *m/z* 417 (Fig. 3a, b). The accurate mass spectrum of compound 1 displayed an ion peak at *m/z* 418.2490, and the estimated composition of the protonated molecular formula was C_26_H_32_N_3_O_2_ (calc 418.2489 (0.1 mDa)). The ^13^C-NMR spectra indicated the existence of another carbonyl (*δ*_C_ 174.4 ppm) in addition to the amide portion of the LSD. Two methylene signals [(*δ*_H_ 1.10 ppm, *δ*_C_ 10.1 ppm), (*δ*_H_ 1.19 ppm, *δ*_C_ 10.1 ppm)] and one methine signal (*δ*_H_ 2.50 ppm, *δ*_C_ 14.4 ppm) were also observed, and 2D NMR correlations revealed the presence of cyclopropanecarbonyl at the N1 position of LSD (Fig. 4). The ^1^H-NMR and ^13^C-NMR shift values of compound **1** were almost the same as those of 1cP-LSD, except for positions 5 and 7. The molecular weight of compound **1** was 26 more than that of 1cP-LSD, and the presence of a partial structure [CH = CH_2_ (*δ*_H_ 6.01 ppm, *δ*_C_ 134.7 ppm), CH = CH_2_ (*δ*_H_ 5.28, 5.35 ppm, *δ*_C_ 119.9 ppm)], which appeared to be a terminal olefin, suggested the presence of an allyl group (Tables 1, 2). The methyl group at N6 was not observed, and correlations were observed between the methylene protons of the allyl group and the carbons at positions 5 and 7, as shown in Fig. 4, indicating that there is an allyl group at position 6 rather than a methyl group. Thus, the structure of compound **1** was determined as 4-(cyclopropanecarbonyl)-*N*,*N*-diethyl-7-(prop-2-en-1-yl)-4,6,6a,7,8,9-hexahydroindolo[4,3-*fg*]quinoline-9-carboxamide (1cP-AL-LAD (**1**)), as shown in Fig. 1.

**Table 1:** ^13^C-NMR data for LSD, 1cP-AL-LAD, 1cP-MIPLA, 1V-LSD, 1cP-LSD and 1B-LSD

No	LSD^a)^	1cP-AL-LAD	1cP-MIPLA	1V-LSD	1cP-LSD^b)^	1B-LSD^b)^
2	120.0	120.7	120.7	120.7	120.7	121.6
3	110.2	118.3	118.1	118.0	118.0	118.0
4	27.9	27.4	27.5	27.4	27.5	27.5
5	64.8	60.9	63.9	63.8	63.8	63.8
7	57.0	52.7	56.1	56.7	56.7	56.7
8	40.6	40.7	41.2	40.6	40.6	40.6
9	119.5	122.2	121.6	121.6	121.6	121.6
10	137.7	136.6	135.9	136.0	136.1	136.0
11	128.3	129.5	129.1	129.1	129.0	129.1
12	112.7	118.2	118.1	118.2	118.0	118.2
13	123.7	127.1	127.2	127.2	127.2	127.2
14	111.0	116.6	116.6	116.6	116.6	116.6
15	135.7	135.2	135.3	135.2	135.3	135.2
16	127.4	129.5	129.4	129.3	129.4	129.3
17	173.9	173.9	173.8	173.6	173.6	173.7
19	41.9	41.9	–	42.0	42.0	42.0
20	13.3	13.3	–	13.3	13.3	13.3
21	43.8	43.8	–	43.8	43.8	43.8
22	15.1	15.1	–	15.1	15.1	15.1
1-N-COCH(CH_2_)_2_	–	174.4	174.4	–	174.4	–
1-N-COCH(CH_2_)_2_	–	14.4	14.4	–	14.4	–
1-N-COCH(CH_2_)_2_	–	10.1	10.1	–	10.1	–
1-N-COCH(CH_2_)_2_	–	10.1	10.1	–	10.1	–
6-N-CH_3_	44.0	–	43.8	43.8	43.8	43.8
6-N-CH_2_CH = CH_2_	–	58.0	–	–	–	–
6-N-CH_2_CH = CH_2_	–	134.7	–	–	–	–
6-N-CH_2_CH = CH_2_	–	119.9	–	–	–	–
18-N-CH_3_	–	–	29.1	–	–	–
18-N–CH(CH_3_)_2_	–	–	46.2	–	–	–
18-N–CH(CH_3_)_2_	–	–	19.5	–	–	–
	–	–	19.5	–	–	–
1-N-COCH_2_CH_2_CH_2_CH_3_	–	–	–	173.6	–	–
1-N-COCH_2_CH_2_CH_2_CH_3_	–	–	–	36.2	–	–
1-N-COCH_2_CH_2_CH_2_CH_3_	–	–	–	28.0	–	–
1-N-COCH_2_CH_2_CH_2_CH_3_	–	–	–	23.4	–	–
1-N-COCH_2_CH_2_CH_2_CH_3_	–	–	–	14.2	–	–
1-N-COCH_2_CH_2_CH_3_	–	–	–	–	–	170.3
1-N-COCH_2_CH_2_CH_3_	–	–	–	–	–	38.3
1-N-COCH_2_CH_2_CH_3_	–	–	–	–	–	19.2
1-N-COCH_2_CH_2_CH_3_	–	–	–	–	–	14.0

^*a)*^ Ref. 9

^b^^*)*^ Ref. 10

**Table 2:** ^1^H-NMR data for LSD, 1cP-AL-LAD, 1cP-MIPLA, 1V-LSD, 1cP-LSD and 1B-LSD

No	LSD^a)^	1cP-AL-LAD	1cP-MIPLA	1V-LSD	1cP-LSD^b)^	1B-LSD^b)^
2	6.95, 1H, d, *J* = 1.4 Hz	7.70, 1H, d, *J* = 2.1 Hz	7.70, 1H, s-like	7.49, 1H, d, *J* = 7.8 Hz	7.71, 1H, d, *J* = 2.1 Hz	7.47, 1H, s
4	2.65, 1H, m	2.60, 1H, ddd, *J* = 2.1, 11.7, 15.1 Hz	2.59, 1H, m, overlapped	2.57, 1H, ddd, *J* = 1.8, 11.9, 14.2 Hz	2.60, 1H, m, overlapped	2.56, 1H, m
	3.57, 1H, ddd, *J* = 6.5, 14.5, 19.3 Hz	3.63, 1H, dd, *J* = 4.8, 15.1 Hz	3.61, 1H, m	3.59, 1H, dd, *J* = 5.5, 15.1 Hz	3.62, 1H, dd, *J* = 5.5, 15.2 Hz	3.59, 1H, dd, *J* = 5.5, 15.1 Hz
5	3.20, 1H, m	3.45, 1H, m	3.19, 1H, m	3.19, 1H, m	3.18, 1H, m	3.17, 1H, m
7	2.76, 1H, t-like, *J* = 11.1 Hz	2.67, 1H, t-like, *J* = 11.0 Hz	2.68, 1H, t-like, *J* = 11.0 Hz	2.73, 1H, t-like, *J* = 11.0 Hz	2.73, 1H, t, *J* = 11.0 Hz	2.72, 1H, t, *J* = 11.0 Hz
	3.07, 1H, dd, *J* = 4.8, 11.0 Hz	3.21, 1H, m	3.13, 1H, dd-like, *J* = 4.6, 11.0 Hz	3.10, 1H, dd-like, *J* = 4.6, 11.4 Hz	3.10, 1H, dd-like, *J* = 4.1, 11.4 Hz	3.09, 1H, dd-like, *J* = 4.1, 11.4 Hz
8	3.95, 1H, m	3.89, 1H, m	3.90, 1H, m	3.96, 1H, m	3.97, 1H, m	3.96, 1H, m
9	6.30, 1H, s-like	6.39, 1H, s-like	6.42, 1H, s-like	6.38, 1H, s-like	6.37, 1H, s-like	6.38, 1H, s-like
12	7.11, 1H, d, *J* = 6.9 Hz	7.39, 1H, d, *J* = 7.6 Hz	7.41, 1H, t-like, *J* = 7.8 Hz	7.41, 1H, d, *J* = 7.8 Hz	7.40, 1H, d, *J* = 7.6 Hz	7.40, 1H, d, *J* = 7.3 Hz
13	7.07, 1H, t-like, *J* = 7.6 Hz	7.29, 1H, t-like, *J* = 7.6 Hz	7.29, 1H, t-like, *J* = 7.8 Hz	7.30, 1H, t-like, *J* = 7.8 Hz	7.29, 1H, t-like, *J* = 7.6 Hz	7.30, 1H, t-like, *J* = 7.8 Hz
14	7.18, 1H, d, *J* = 8.3 Hz	8.03, 1H, d, *J* = 8.3 Hz	8.03, 1H, d, *J* = 8.3 Hz	8.05, 1H, brs	8.03, 1H, d, *J* = 8.3 Hz	8.05, 1H, br
19	3.44, 2H, m	3.41, 1H, m, overlapped	–	3.41, 1H, m	3.41, 1H, m	3.40, 1H, m
		3.44, 1H, m, overlapped	–	3.47, 1H, m	3.47, 1H, m	3.47, 1H, m
20	1.17, 3H, t, *J* = 7.3 Hz	1.16, 3H, t, *J* = 7.3 Hz	–	1.17, 3H, t, *J* = 7.3 Hz	1.17, 3H, t, *J* = 7.3 Hz	1.17, 3H, t, *J* = 7.3 Hz
21	3.55, 2H, m, overlapped	3.53, 2H, m, overlapped	–	3.55, 2H, m, overlapped	3.55, 2H, m, overlapped	3.55, 2H, m
22	1.29, 3H, t, *J* = 7.3 Hz	1.28, 3H, t, *J* = 7.3 Hz	–	1.29, 3H, t, *J* = 7.3 Hz	1.30, 3H, t, *J* = 6.9 Hz	1.29, 3H, t, *J* = 7.3 Hz
1-N-COCH(CH_2_)_2_	–	2.50, 1H, m	2.50, 1H, m	–	2.51, 1H, m	–
1-N-COCH(CH_2_)_2_	–	1.10, 2H, m	1.10, 2H, m	–	1.11, 2H, m	–
1-N-COCH(CH_2_)_2_	–	1.19, 2H, m	1.19, 2H, m	–	1.19, 2H, m	–
6-N-CH_3_	2.60, 3H, s	–	2.61, 3H, s	2.61, 3H, s	2.61, 3H, s	2.60, 3H, s
6-N-CH_2_CH = CH_2_	–	3.23, 1H, dd, *J* = 9.0, 14.5 Hz	–	–	–	–
	–	3.76, 1H, dd, *J* = 4.8, 14.4 Hz	–	–	–	–
6-N-CH_2_CH = CH_2_	–	6.01, 1H, m	–	–	–	–
6-N-CH_2_CH = CH_2_	–	5.28, 1H, d, *J* = 10.0 Hz	–	–	–	–
	–	5.35, 1H, d, *J* = 16.5 Hz		–	–	–
18-N-CH_3_	–	–	3.05, 3H, s	–	–	–
18-N–CH(CH_3_)_2_	–	–	4.82, 1H, m	–	–	–
18-N–CH(CH_3_)_2_	–	–	1.15, 3H, d, *J* = 6.9 Hz	–	–	–
	–	–	1.19, 3H, d, *J* = 6.9 Hz	–	–	–
1-N-COCH_2_CH_2_CH_2_CH_3_	–	–	–	2.97, 2H, t, *J* = 7.3 Hz	–	–
1-N-COCH_2_CH_2_CH_2_CH_3_	–	–	–	1.78, 2H, dddd, *J* = 7.3, 7.8, 7.8, 7.8 Hz	–	–
1-N-COCH_2_CH_2_CH_2_CH_3_	–	–	–	1.48, 2H, dddd, *J* = 7.3, 7.3, 7.8, 15.1 Hz	–	–
1-N-COCH_2_CH_2_CH_2_CH_3_	–	–	–	0.99, 3H, t, *J* = 7.3 Hz	–	–
1-N-COCH_2_CH_2_CH_3_					–	2.95, 2H, t, *J* = 7.3 Hz
1-N-COCH_2_CH_2_CH_3_	–	–	–	–	–	1.83, 2H, ddd, *J* = 7.3, 14.6, 14.6 Hz
1-N-COCH_2_CH_2_CH_3_	–	–	–	–	–	1.07, 3H, t, *J* = 7.3 Hz

^*a*^ Ref. 9

### Analysis of sheet product B

**Fig. 5:**
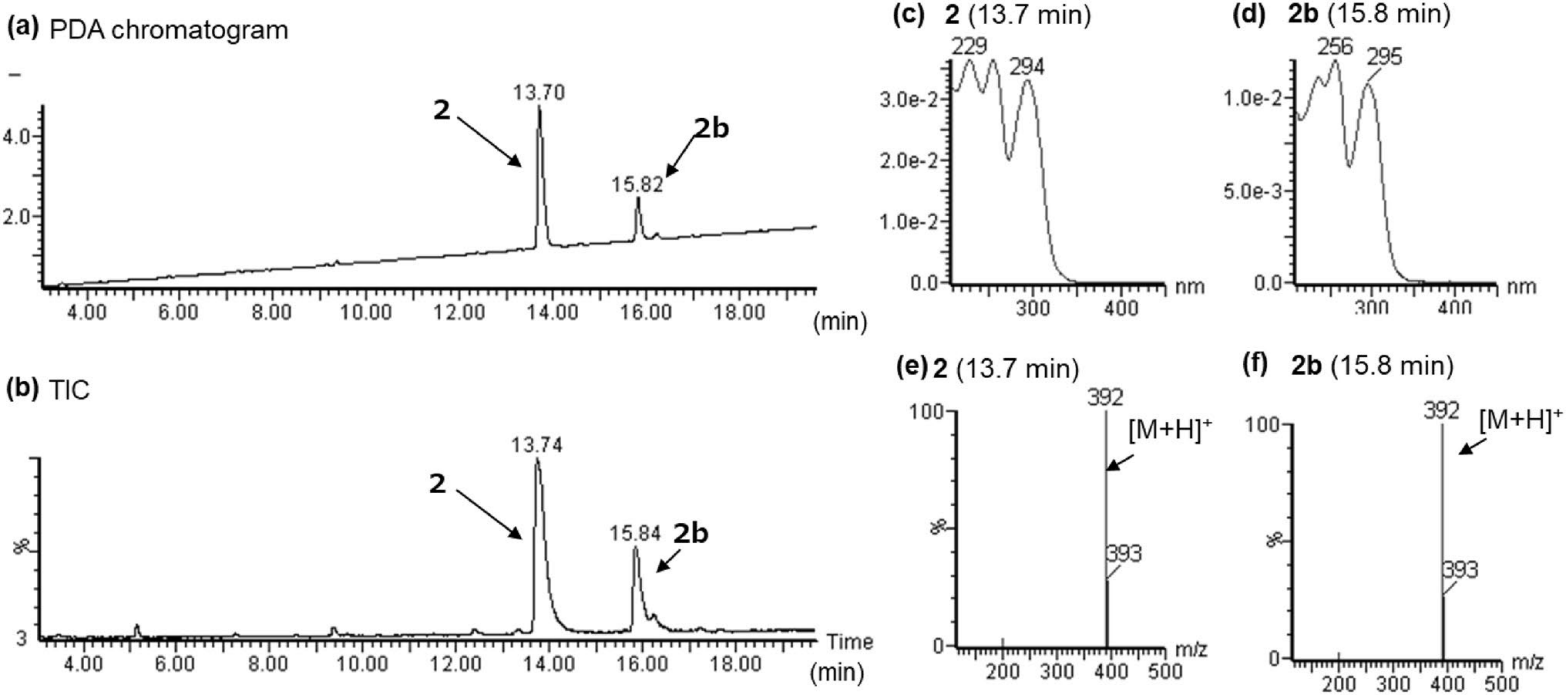
LC–PDA–MS analysis of sheet B; PDA chromatogram **a**, TIC **b**, and UV spectra of peak 2 **c** and of peak 2b **d**, and ESI mass spectra of peak 2 **e** and of peak 2b **f**

**Fig. 6:**
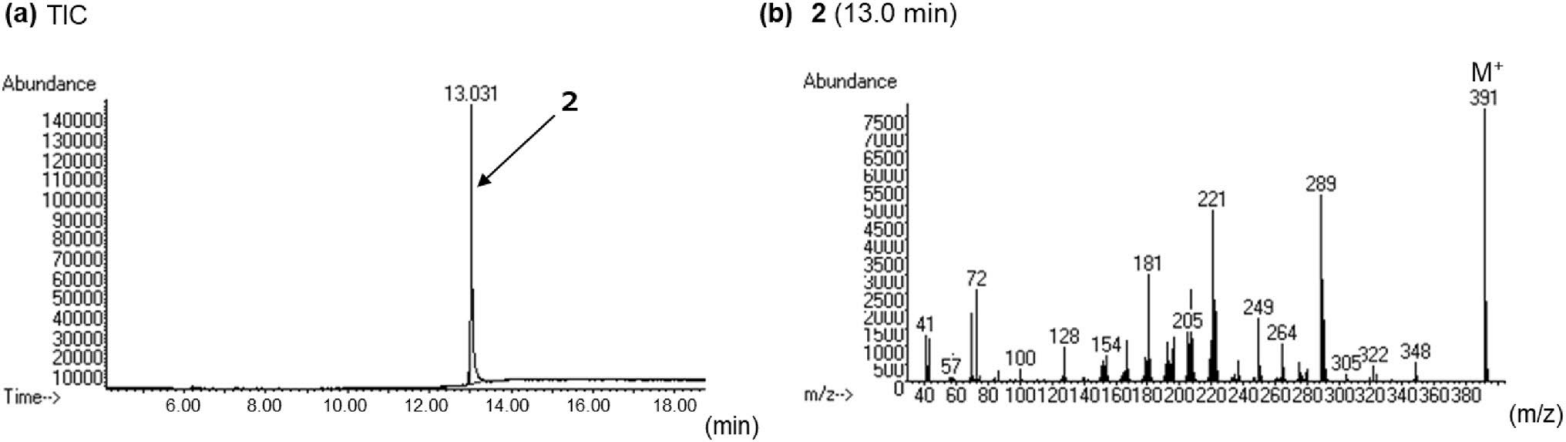
GC–MS analysis of sheet B; TIC **a**, EI mass of peak 2 **b**

In the LC–PDA–MS analysis of product B, a peak of compound 2 was detected at 13.7 min with a protonated molecular ion ([M + H]^+^) at *m/z* 392 (Fig. 5a, b). In the GC–MS analysis, a peak at 13.0 min revealed a molecular ion ([M]^+^) at *m/z* 391 (Fig. 6a, b). The accurate mass spectrum of compound 2 revealed an ion peak at *m/z* 392.2333, and the estimated composition of the protonated molecular formula was C_24_H_30_N_3_O_2_ [calc 392.2333 (0 mDa)]. The NMR spectroscopic data showed the presence of another carbonyl (*δ*_C_ 174.4 ppm), two methylenes [(*δ*_H_ 1.10 ppm, *δ*_C_ 10.1 ppm), (*δ*_H_ 1.19 ppm, *δ*_C_ 10.1 ppm)] and one methine (*δ*_H_ 2.50 ppm, *δ*_C_ 14.4 ppm) in addition to the LSD moiety, and they were correlated in 2D NMR spectrum, as shown in Fig. 4. Thus, compound 3 is presumed to be cyclopropionylation at the N1 position, as is 1cP-AL-LAD (1). Two doublet methyl [(*δ*_H_ 1.15 ppm, *δ*_C_ 19.5 ppm), (*δ*_H_ 1.19 ppm, *δ*_C_ 19.5 ppm)] and methine (*δ*_H_ 4.82 ppm, *δ*_C_ 46.2 ppm) signals were observed, suggesting the presence of isopropyl groups (Table 1 and 2). One singlet methyl (*δ*_H_ 3.05 ppm, *δ*_C_ 29.1 ppm) was observed, but no signal derived from the diethyl group at the N18 of LSD was observed. The correlation shown in Fig. 4 indicates that the N18 position of LSD is not a diethyl group but methyl and isopropyl groups. Thus, the structure of compound 2 was determined as 4-(cyclopropanecarbonyl)-*N*-methyl-*N*-isopropyl-7-methyl-4,6,6a,7,8,9-hexahydroindolo-[4,3-*fg*]quinoline-9-carboxamide (1cP-MIPLA (**2**)), as shown in Fig. 1.

In the ^1^H-NMR and ^13^C-NMR spectra, there are minor signals at positions 7–9 and at the *N*-methyl-*N*-isopropyl moiety. As the ratios of these minor signals were changed in different solvents and the same phenomenon was observed for MIPLA, it was presumed that these minor signals were derived from the rotational isomer of the N18 amide moiety of 1cP-MIPLA.

In the LC–PDA–MS analysis, a peak of 2b with the same *m/z* 392 [M + H]^+^ as 1cP-MIPLA was detected at 15.8 min (Fig. 5b), in addition to the peak of 1cP-MIPLA. The UV spectrum of 2b was not similar to that of 1cP-MIPLA. In addition, the signals presumed to be derived from minor compounds other than 1cP-MIPLA were also observed in the ^1^H-NMR and ^13^C-NMR spectra. It has been reported that the epimerization of LSD at position 8 occurs to produce *iso*-LSD [13, 14]. As epimerization at position 8 also occurs during the synthesis of LSD analogs [15], this minor compound was presumed to be *iso*-1cP-MIPLA.

### Analysis of sheet product C

**Fig. 7:**
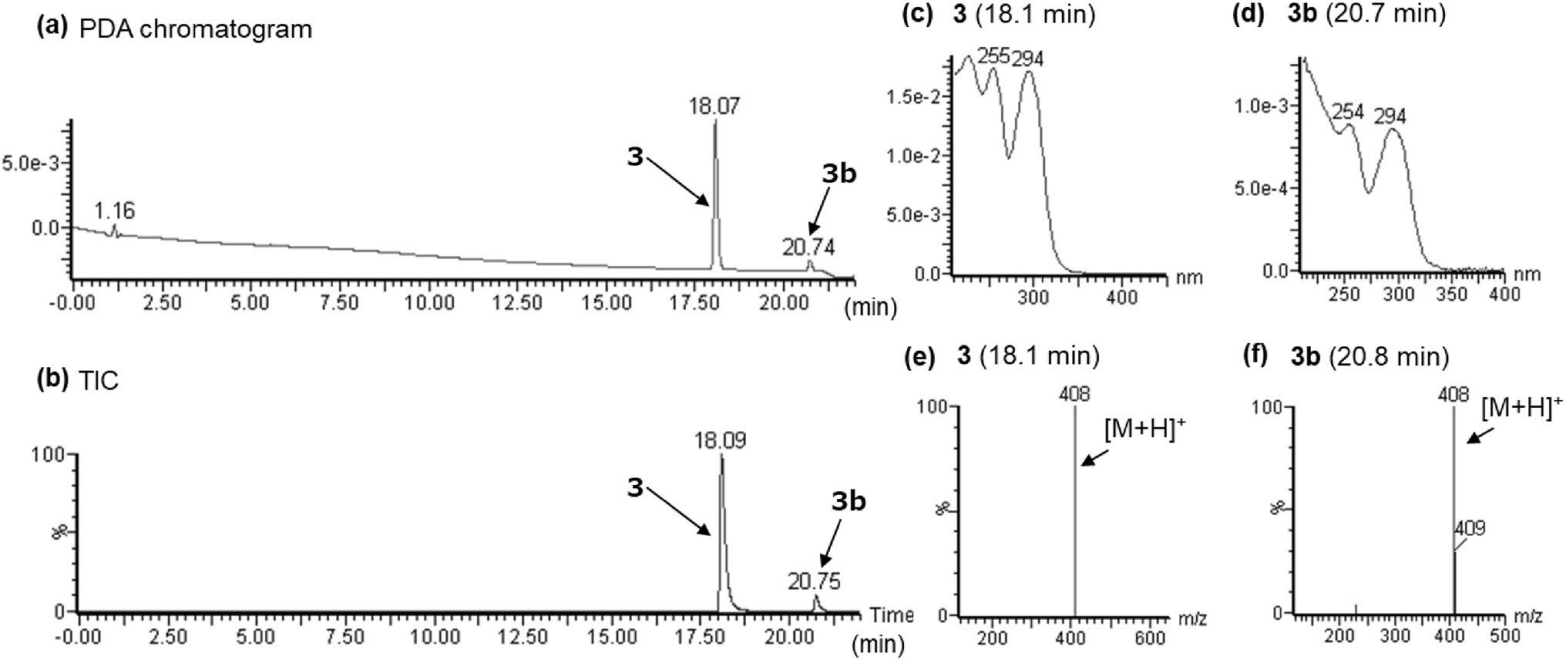
LC–PDA–MS analysis of sheet C; PDA chromatogram **a**, TIC **b** and UV spectra of peak 3 **c** and of peak 3b **d**, and ESI mass spectra of peak 3 **e** and of peak 3b **f**

**Fig. 8:**
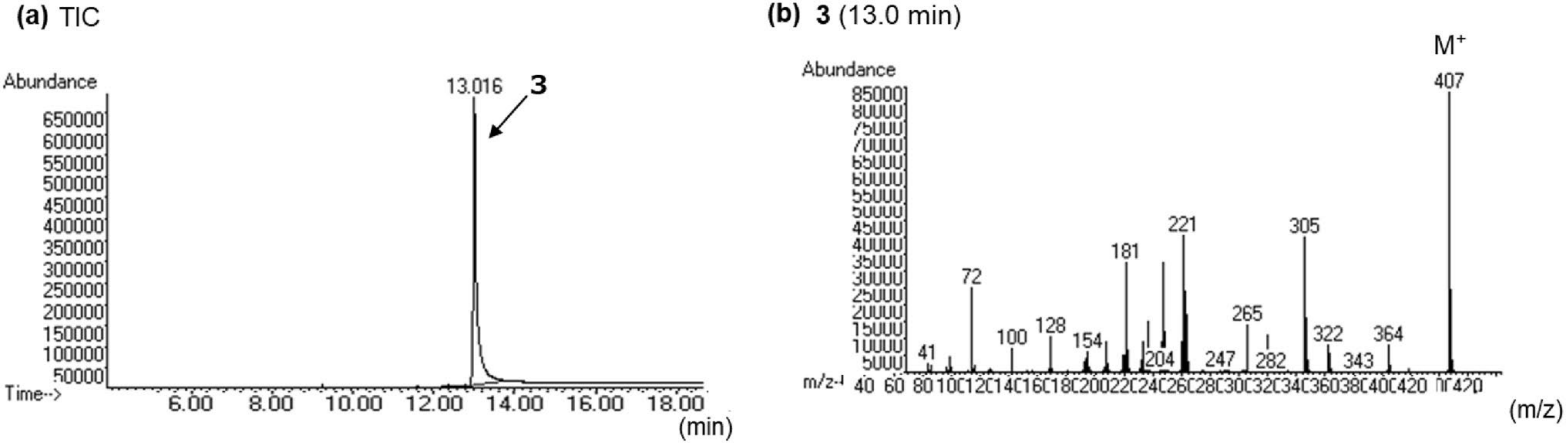
GC–MS analysis of sheet C; TIC **a**, EI mass of peak 3 **b**

In the LC–PDA–MS analysis of product C, a peak of compound **3** was detected at 18.0 min with a protonated molecular ion ([M + H]^+^) at *m/z* 408 (Fig. 7a, b). In the GC–MS analysis, the peak at 13.0 min revealed a molecular ion ([M]^+^) at *m/z* 407 (Fig. 8a, b). The accurate mass spectrum of compound **3** contained an ion peak at *m/z* 408.2647, and the estimated composition of the protonated molecule formula was C_26_H_32_N_3_O_2_ (calc 408.2646 (0.1 mDa)). The NMR spectroscopic data showed the presence of another carbonyl (*δ*_C_ 173.8 ppm), three methylenes [(*δ*_H_ 2.97 ppm, *δ*_C_ 36.2 ppm), (*δ*_H_ 1.78 ppm, *δ*_C_ 28.0 ppm), (*δ*_H_ 1.48 ppm, *δ*_C_ 23.4 ppm)], and one methyl (*δ*_H_ 0.99 ppm, *δ*_C_ 14.2 ppm) in addition to the LSD moiety, and they were correlated in the 2D NMR spectra, as shown in Fig. 4. The data suggested the presence of a pentanoyl group. The chemical shift values of ^1^H-NMR and ^13^C-NMR spectra were in good agreement with those of 1B-LSD (Tables 1 and 2). As the estimated compositional formula of compound **3** contains more CH_2_ than that of 1B-LSD, and the correlation was observed, as shown in Fig. 4, the N1 position of LSD was presumed to be pentanoylated. Thus, the structure of compound 3 was determined as *N,N*-diethyl-7-methyl-4-pentanoyl-4,6,6a,7,8,9-hexahydroindolo[4,3-*fg*]quinoline-9-carboxamide (1V-LSD (3)) [12], as shown in Fig. 1.

In the LC–PDA–MS analysis, a peak of 3b with the same *m/z* 392 [M + H]^+^ as 1V-LSD was detected at 20.7 min (Fig. 7b) in addition to the peak of 1V-LSD. The UV spectrum of 3b was not similar to that of 1V-LSD. Moreover, the signals presumed to be derived from minor compound other than 1V-LSD were also observed in the ^1^H-NMR and ^13^C-NMR spectra. This minor compound was estimated to be *iso*-1V-LSD, C8-epimerized 1V-LSD.

### Analysis of sheet product D

In the LC–PDA–MS analysis of sheet D, a peak of compound **4** was detected at 6.8 min with a protonated molecular ion ([M + H]^+^) at *m/z* 336 (Fig. S1). In the GC–MS analysis, a peak at 11.4 min showed a molecular ion ([M]^+^) at *m/z* 335 (Fig. S2a and S2b). After comparing the data from LC–PDA–MS and GC–MS analyses with those of the authentic compound, this compound was identified as LSZ (4) [5, 16], a compound in which the diethyl moiety at position N18 of LSD has been converted to 2,4-dimethylazetidin.

## Discussion

Based on the structure of LSD, the detected compound, 1cP-AL-LAD, was speculated to be converted at the positions at N1 and N6, and 1cP-MIPLA be converted at the positions at N1 and N18. This is the first report in which LSD analogs that have been converted at multiple positions have been detected in sheet products in Japan.

It has been reported that N1-acylated LSD analogs in methanol solutions are partially deacylated during GC–MS analysis [9, 10, 15]. In this study, we observed the partial deacylation of 1V-LSD, but not of 1cP-AL-LAD and 1cP-MIPLA under the same conditions. The reason that 1cP-AL-LAD and 1cP-MIPLA were not deacylated could also be conceived, because they were also modified with N6 or N18. However, Simon et al. reported that 1P-AL-LAD, converted at the N1 and N6 positions, was partially deacylated to AL-LAD during GC–MS analysis [15]. Therefore, 1cP-AL-LAD and 1cP-MIPLA may also be partially deacylated depending on the GC–MS analysis conditions. Halberstadt et al*.* [11] reported that high concentrations of LSD were detected in the plasma of rats after the subcutaneous administration of ALD-52 and 1P-LSD. 1V-LSD might be deacylated in vivo and may function as a prodrug of LSD. The products containing these prodrug-type compounds might cause health damage, similar to LSD.

It has been reported that under alkaline conditions, approximately 10% of LSD is epimerized to *iso*-LSD after prolonged heat exposure [13, 14]. In addition, the C8-epimerization of 1P-AL-LAD during GC–MS analysis was reported by Simon et al. [15]. In this study, the presence of *iso*-1cP-MIPLA and *iso*-1V-LSD was suggested, and C8-epimerization of other LSD analogs may also occur. The metabolic pathway and biological activities of 1cP-AL-LAD and 1cP-MIPLA have not been reported.

## Conclusions

This is the first report showing that LSD analogs that were converted at multiple positions have been detected in sheet products in Japan. In this report, we analyzed LSD analogs in four sheet products. As a results, we identified three compounds as 1cP-AL-LAD, 1cP-MIPLA, and 1V-LSD by NMR analyses, one compound as LSZ by comparison of the data with the authentic compound. The possibility of deacylation in vivo and the conversion into AL-LAD or MIPLA should be further investigated. In ﻿﻿addition﻿,﻿ there﻿ are﻿ concerns about the future distribution of sheet drug products containing new LSD analogs. Therefore, the continuous monitoring of newly detected compounds in sheet products is important.

## Supplementary Information

Below is the link to the electronic supplementary material.

Supplementary file1 (DOCX 238 KB)

## Data Availability

All data generated or analyzed during this study are included in this published article and its supplementary material files.
