# Supplementary figures and images for: Identification of LSD analogs, 1cP-AL-LAD, 1cP-MIPLA, 1V-LSD and LSZ in sheet products
Source: Forensic Toxicol. 2023 Feb 21;41(2):294–303. doi: 10.1007/s11419-023-00661-1 (PMC10310582; doi:10.1007/s11419-023-00661-1)

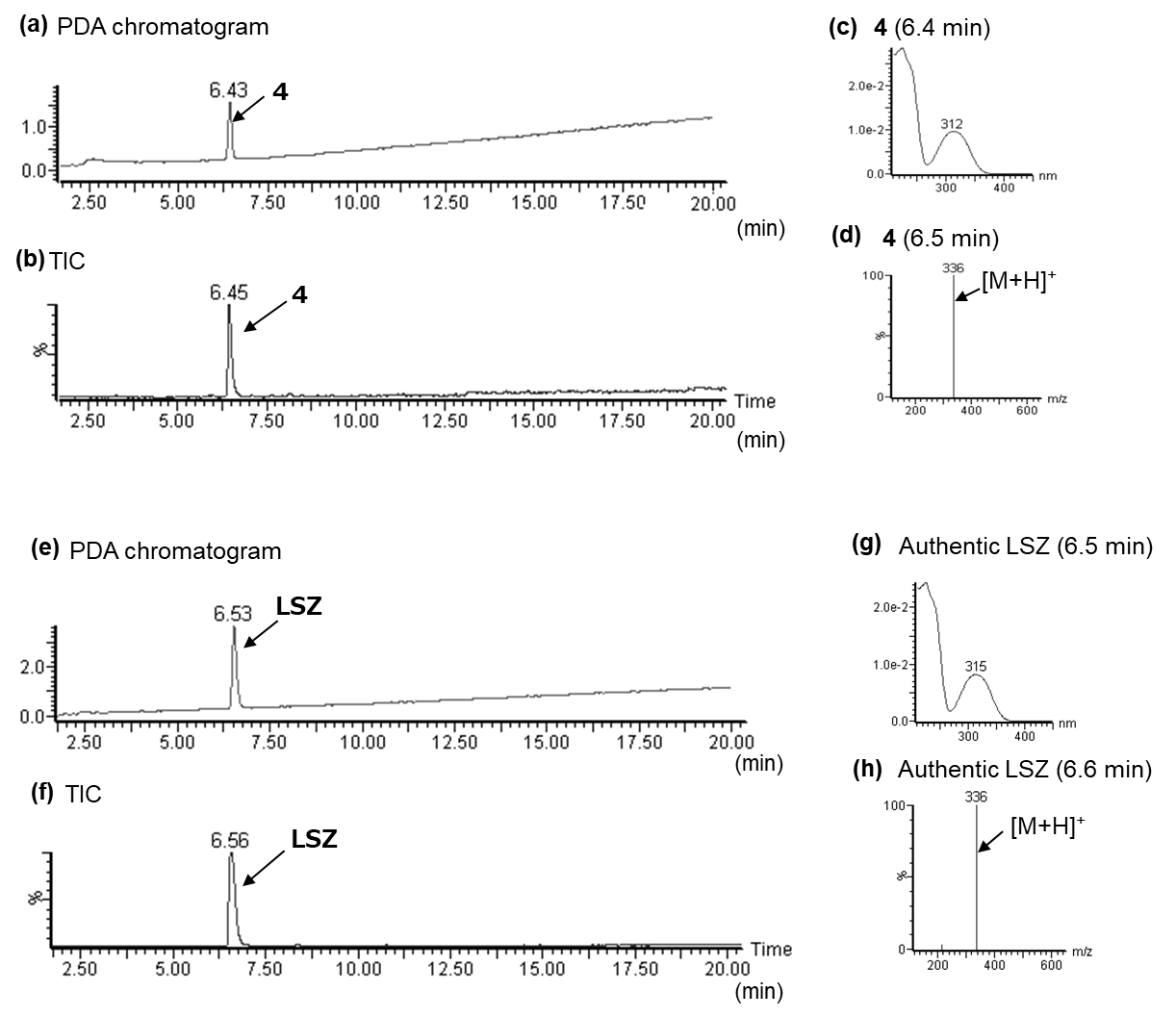


Fig. S1


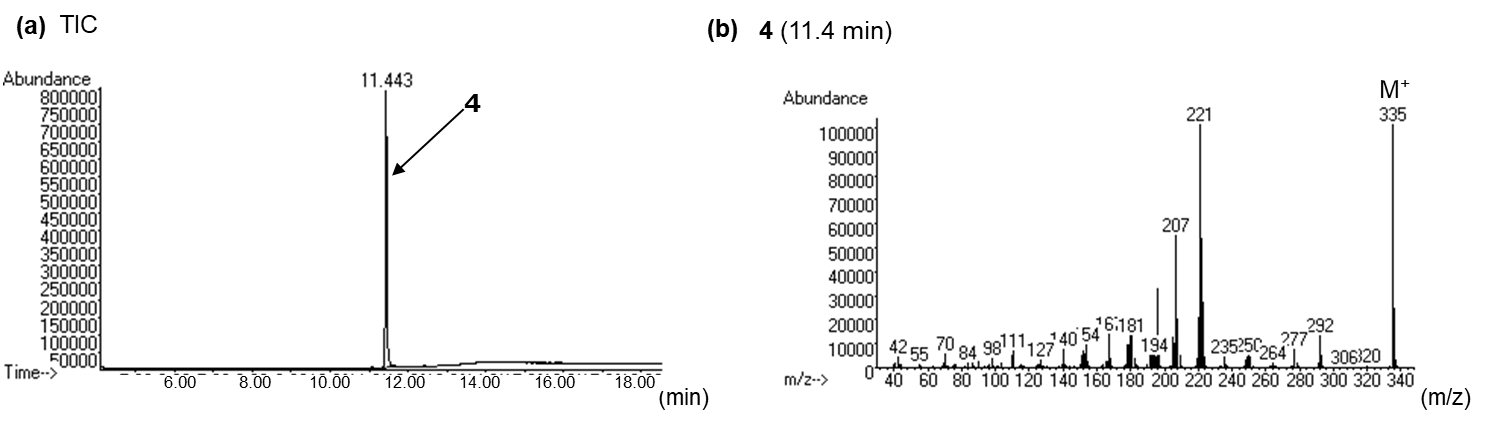


Fig. S2

Table S1 NMR data of LSZ

Supplement: Supplementary file 1 — Supplementary file1 (DOCX 238 KB) [file 11419_2023_661_MOESM1_ESM.docx]
